# Management of single brain metastasis: a practice guideline

**DOI:** 10.3747/co.2007.129

**Published:** 2007-08

**Authors:** A. Mintz, J. Perry, K. Spithoff, A. Chambers, N. Laperriere

**Affiliations:** * University of Pittsburgh, Department of Neurological Surgery, Pittsburgh, Pennsylvania, U.S.A; † Toronto–Sunnybrook Regional Cancer Centre, Toronto, Ontario.; ‡ Cancer Care Ontario, Program in Evidence-Based Care, McMaster University, Hamilton, Ontario.; § Princess Margaret Hospital, Toronto, Ontario

**Keywords:** Brain metastasis, surgery, radiotherapy, radiosurgery, systematic review, practice guideline

## Abstract

**Questions:**

Should patients with confirmed single brain metastasis undergo surgical resection?

Should patients with single brain metastasis undergoing surgical resection receive adjuvant whole-brain radiation therapy (wbrt)?

What is the role of stereotactic radiosurgery (srs) in the management of patients with single brain metastasis?

**Perspectives:**

Approximately 15%–30% of patients with cancer will develop cerebral metastases over the course of their disease. Patients identified as having single brain metastasis generally undergo more aggressive treatment than do those with multiple metastases; however, in the province of Ontario, management of patients with single brain metastasis varies. Given that conflicting evidence has been reported, the Neuro-oncology Disease Site Group (dsg) of the Cancer Care Ontario Program in Evidence-based Care felt that a systematic review of the evidence and a practice guideline were warranted.

**Outcomes:**

Outcomes of interest were survival, local control of disease, quality of life, and adverse effects.

**Methodology:**

The medline, cancerlit, embase, and Cochrane Library databases and abstracts published in the proceedings of the annual meetings of the American Society of Clinical Oncology (1997–2005) and American Society for Therapeutic Radiology and Oncology (1998–2004) were systematically searched for relevant evidence. The review included fully published reports or abstracts of randomized controlled trials (rcts), nonrandomized prospective studies, and retrospective studies.

The present systematic review and practice guideline has been reviewed and approved by the Neuro-oncology dsg, which comprises medical and radiation oncologists, surgeons, neurologists, a nurse, and a patient representative. External review by Ontario practitioners was obtained through an electronic survey. Final approval of the guideline report was obtained from the Report Approval Panel and the Neuro-oncology dsg.

**Results:**

**Quality of Evidence**

The literature search found three rcts that compared surgical resection plus wbrt with wbrt alone. In addition, a Cochrane review, including a meta-analysis of published data from those three rcts, was obtained.

One rct compared surgical resection plus wbrt with surgical resection alone. One rct compared wbrt plus srs with wbrt alone. Evidence comparing srs with surgical resection or examining srs with or without wbrt was limited to prospective case series and retrospective studies.

**Benefits**

Two of three rcts reported a significant survival benefit for patients who underwent surgical resection as compared with those who received wbrt alone. Pooled results of the three rcts indicated no significant difference in survival or likelihood of dying from neurologic causes; however, significant heterogeneity was detected between the trials. The rct that compared surgical resection plus wbrt with surgical resection alone reported no significant difference in overall survival or length of functional independence; however, tumour recurrence at the site of the metastasis and anywhere in the brain was less frequent in patients who received wbrt as compared with patients in the observation group. In addition, patients who received wbrt were less likely to die from neurologic causes.

Results of the rct that compared wbrt plus srs with wbrt alone indicated a significant improvement in median survival in patients who received srs. No quality evidence compares the efficacy of srs with surgical resection or examines the question of whether patients who receive srs should also receive wbrt.

**Harms**

Pooled results of the three rcts that examined surgical resection indicated no significant difference in adverse effects between groups. Postoperative complications included respiratory problems, intracerebral hemorrhage, and infection. One rct reported no significant difference in adverse effects between patients who received wbrt plus srs and those who received wbrt alone.

**Practice Guideline:**

**Target Population**

The recommendations that follow apply to adults with confirmed cancer and a single brain metastasis. This practice guideline does not apply to patients with metastatic lymphoma, small-cell lung cancer, germ-cell tumour, leukemia, or sarcoma.

**Recommendations**

Surgical excision should be considered for patients with good performance status, minimal or no evidence of extracranial disease, and a surgically accessible single brain metastasis amenable to complete excision. Because treatment in cases of single brain metastasis is considered palliative, invasive local treatments must be individualized. Patients with lesions requiring emergency decompression because of intracranial hypertension were excluded from the rcts, but should be considered candidates for surgery.

To reduce the risk of tumour recurrence for patients who have undergone resection of a single brain metastasis, postoperative wbrt should be considered. The optimal dose and fractionation schedule for wbrt is 3000 cGy in 10 fractions or 2000 cGy in 5 fractions.

As an alternative to surgical resection, wbrt followed by srs boost should be considered for patients with single brain metastasis. The evidence is insufficient to recommend srs alone as a single-modality therapy.

**Qualifying Statements**

No high-quality data are available regarding the choice of surgery versus radiosurgery for single brain metastasis. In general, the size and location of the metastasis determine the optimal approach.

The standard wbrt regimen for management of patients with single brain metastasis in the United States is 3000 cGy in 10 fractions, and this treatment is usually the standard arm in randomized studies of radiation in patients with brain metastases. Based solely on evidence, the understanding that no reason exists to choose 3000 cGy in 10 fractions over 2000 cGy in 5 fractions is correct; however, fraction size is believed to be important, and therefore 300 cGy daily (3000/10) is believed to be associated with fewer long-term neurocognitive effects than 400 cGy daily (2000/5) in the occasional long-term survivor. For that reason, many radiation oncologists in Ontario prefer 3000 cGy in 10 fractions. No data exist to either support or refute that preference; therefore, finding a resolution to this issue is not currently possible. The Neuro-oncology dsg will update the recommendations as new evidence becomes available.

## 1. INTRODUCTION

### Questions

Should patients with confirmed single brain metastasis undergo surgical resection?

Should patients with single brain metastasis undergoing surgical resection receive adjuvant whole brain radiation therapy (wbrt)?

What is the role of stereotactic radiosurgery (srs) in the management of patients with single brain metastasis?

## 2. CHOICE OF TOPIC AND RATIONALE

Cerebral metastases occur in 15%–30% of cancer patients during the course of their disease^1^–^3^. Approximately half of these patients have single metastasis as shown by computed tomography (ct) imaging^2^–^4^. Patients with single metastasis tend to undergo more aggressive therapy than do those with multiple metastases; treatment guidelines should therefore be specific to this patient group.

Because the distinctions between intracranial primary and metastatic cancer and between single and multiple metastases frequently determine choice of treatment, care must be taken in the initial diagnosis of a suspected metastasis. Contrast-enhanced ct imaging or magnetic resonance imaging (mri) are the standard diagnostic tests for individuals suspected of intracranial primary or metastatic cancer. In individuals that appear to have a single metastasis and in whom the primary tumour site is controlled or unknown, high-dose contrast imaging studies are appropriate. These studies may be accomplished with iodinated contrast and a repeat ct scan. Alternatively, high-dose contrast gadolinium-enhanced mri may be used, because it has demonstrated increased sensitivity in detecting smaller lesions. However, in several studies using mri, between 2% and 11% of patients were misdiagnosed as having single brain metastasis^5^,^6^. Surgical resection or stereotactic biopsy should be used if a solitary lesion with characteristics of a cancer is seen with no known primary to establish tissue diagnosis before other treatments commence.

In Ontario, management of patients with suspected single brain metastasis currently varies. The Neuro-oncology Disease Site Group (dsg), which represents 9 regional cancer centres, conducted an informal poll to establish the current practice in Ontario for the treatment of patients with single brain metastasis. The findings were categorized by patient prognosis (good vs. poor) based on the Karnofsky performance score (kps) and the status of the underlying primary disease. However, it should be noted that no formal criteria for prognosis have been established.

Patients with a “good” prognosis would generally undergo resection by craniotomy, followed by wbrt 3000 cGy in 10 fractions, although patients treated at 2 regional cancer centres (rccs) receive 2000 cGy in 5 fractions, and at 2 other rccs, the dose varies. At some rccs, patients receive boost radiation or srs if the lesion is unresectable.

At most rccs, patients with a “poor” prognosis do not undergo resection. At 7 rccs, patients receive 2000 cGy in 5 fractions, but at the other 2 centers, the dose varies depending on the pathology. Patients are referred for surgical consideration based both on tumour-specific factors (location, size, or degree of mass effect) and on patient-specific factors (age, co-morbid medical conditions, or extracranial disease). The decision to operate is also based on the foregoing factors, with local physician referral patterns and individual judgments about the patient, rather than rcc-specific guidelines, being the rule.

Based on the conflicting results from the three randomized trials of surgery and radiation therapy as compared with radiation therapy alone, the increasing use of srs, and the variation in treatment across rccs in Ontario, the Neuro-oncology dsg felt that a systematic review and practice guideline were warranted.

## 3. METHODS

### 3.1 Guideline Development

The present practice guideline report was developed by Cancer Care Ontario’s Program in Evidence-based Care (pebc), using the methods of the practice guidelines development cycle^7^. Evidence was selected and reviewed by members of the pebc Neuro-oncology dsg and by methodologists. Members of the Neuro-oncology dsg disclosed potential conflicts of interest. The pebc is editorially independent of Cancer Care Ontario and the Ontario Ministry of Health and Long-Term Care.

This systematic review is a convenient and up-to-date source of the best available evidence on the management of single brain metastasis and is intended to promote evidence-based practice in Ontario, Canada. Because the body of evidence in this review includes mature rct data, the Neuro-oncology dsg provides recommendations.

Final approval of the guideline report was obtained from the Report Approval Panel (rap) and the Neuro-oncology dsg. External review by Ontario practitioners was obtained through an electronic survey.

### 3.2 Literature Search Strategy

The medline (1966 through December 2005), embase (1980 through week 52, 2005), cancerlit (1983 through October 2002), and the Cochrane Library (2005, Issue 4) databases were searched with no language restrictions. “Brain neoplasms” [medical subject heading (mesh)], “brain adj2 metastas#s” (text word), “cerebral adj2 metastas#s” (text word), or “metastatic brain” were combined with “single” or “solitary” used as text words. These search terms were combined with “radiotherapy, adjuvant” (mesh), “combined modality therapy” (mesh), and “radiosurgery” (mesh), and the following text words: “surgery,” “radiation,” “radiotherapy,” and “radiosurgery.” Those terms were then combined with the search terms for the following study designs: practice guidelines, meta-analyses, rcts, clinical trials, cohort studies, and retrospective studies. In addition, the proceedings of major conferences, including the annual meetings of the American Society of Clinical Oncology (1997–2005) and the American Society for Therapeutic Radiology and Oncology (1998–2004), were also searched for reports of new or ongoing trials. Relevant articles and abstracts were selected and reviewed, and the reference lists from those sources were searched for additional trials.

### 3.3 Study Selection Criteria

Articles were selected for inclusion in this systematic review of the evidence if they

- were fully published reports or published abstracts of meta-analyses, systematic reviews, and rcts addressing specific guideline questions. If no studies of those types were available, non-randomized prospective studies and retrospective reviews were eligible for inclusion.
- included these outcomes of interest: survival, local control of disease, quality of life, and adverse effects. Studies had to report data on at least one of these outcomes to be eligible for inclusion.

Articles were excluded from this systematic review of the evidence if they

- were letters or editorials.
- were published in a language other than English.
- were studies of patients with metastatic lymphoma, small-cell lung cancer, germ-cell tumour, leukemia, and sarcoma.
- included patients with multiple brain metastases and did not separately report results for patients with single brain metastasis.

### 3.4 Synthesizing the Evidence

The results from three rcts of surgical resection of single brain metastasis^5^,^6^,^8^ were not pooled because a published meta-analysis using summary data from those three trials was available^9^.

## 4. RESULTS

### 4.1 Literature Search Results

Table I outlines, by question, the type and number of studies included in this practice guideline.

### 4.2 Outcomes

#### 4.2.1 Should Patients with Confirmed Single Brain Metastasis Undergo Surgical Resection?

Three rcts compared surgery plus wbrt with wbrt alone in the treatment of single brain metastasis 5,6,8 (Table II). All three trials required patients to have histologically verified extracranial cancer and radiographic evidence of a surgically resectable single brain metastasis. Patients with certain radiosensitive tumours such as lymphoma and small-cell lung cancer were excluded from all trials. In the trial by Patchell *et al.*^5^, patients were stratified by tumour location, extent of disease, and type of primary tumour. Stratification in the trial by Vecht *et al.*^8^ was by centre, site of extracranial disease, and status of extracranial disease. Mintz *et al.*^6^ stratified patients by type of cancer, size of metastasis, and extent of primary cancer. In all trials, most patients had non-small-cell lung cancer; other primary tumours types included breast, gastrointestinal, genitourinary, and melanoma. All patients randomized to the surgery plus wbrt groups in the trials by Patchell *et al.*^5^ and Vecht *et al.*^8^ underwent surgical resection, but treatment compliance with wbrt was not reported. In the trial by Mintz and colleagues^6^, 6 patients in the surgery plus wbrt group did not receive wbrt and 2 patients did not undergo surgery. In the wbrt alone group, 1 patient did not receive wbrt, and 10 patients underwent a surgical procedure.

Analyses for all three rcts used the intent-to-treat principle.

##### Survival

Two randomized trials demonstrated a significant survival benefit for patients who received surgery plus wbrt as compared with patients who received wbrt alone^5^,^8^, and one randomized trial detected no significant survival difference between the treatment groups^6^. In the trial by Patchell *et al.*^5^, median survival was 9.2 months for patients who received surgery as compared with 3.5 months for patients who received wbrt alone (*p* < 0.01); in the trial by Vecht *et al.*^8^, median survival was 10 months as compared with 6 months (*p* = 0.04). Also in the trial by Vecht *et al.,* the difference in survival was most robust in a subgroup of patients with stable or absent extracranial disease (median survival: 12 months vs. 7 months; *p* = 0.02). No significant survival difference was observed in patients with active extracranial disease (median survival: 5 months in both treatment groups; *p* = 0.88).

In the rct by Mintz *et al.*^6^, median survival was not statistically different between the surgery plus wbrt arm and the wbrt-only arm (5.6 months and 6.3 months respectively, *p* = 0.24). In addition, most patients died within the first year (69.8% in the wbrt arm, 87.8% in the surgery plus wbrt arm). In a univariate Cox proportional hazard model, the systemic extent of primary disease was identified as a major contributing factor and predictor of mortality (relative risk:1.86; *p* = 0.006).

A Cochrane collaboration meta-analysis of the published survival data from the three trials indicated no significant difference in overall survival, with a hazard ratio of 0.74 [95% confidence interval (CI): 0.39 to 1.4; *p* = 0.35]^9^. A high degree of heterogeneity for survival was detected between trials.

##### Neurologic Control of Disease

Local recurrence of disease was reported in only one randomized trial^5^. In the trial by Patchell *et al.,* recurrence or progression at the site of the original metastasis was less frequent in the surgery plus wbrt group than in the wbrt-only group (20% vs. 52%, *p* < 0.02). The median length of time from treatment to recurrence of the brain metastasis was significantly longer in patients who underwent surgery than in patients who received wbrt alone (>59 weeks vs. 21 weeks; *p* < 0.0001).

None of the three randomized trials reported a significant difference between neurologic and systemic causes of death between the treatment groups. Mintz *et al.*^6^ reported that the cause of death was systemic disease in 46% of the surgical group and in 35% of the radiation group (*p* = 0.42). Death from neurologic causes alone was 15% in the surgical group and 28% in the radiation group (*p* = 0.30). The remaining patients died of a combination of neurologic and systemic causes or an unknown cause. Vecht *et al.*^8^ reported no difference in systemic or neurologic causes of death between the treatment groups, with neurologic death being approximately one third in both treatment groups. Patchell *et al.*^5^ reported that 71% of patients in the surgical group and 50% in the wbrt-only group died of systemic causes (*p* = 0.26). The Cochrane meta-analysis indicated that patients who were treated with surgery were somewhat less likely to die from neurologic causes [odds ratio (or): 0.57; 95% ci: 0.29 to 1.10; *p* = 0.09], but this trend was not statistically significant^9^.

##### Quality of Life and Performance Status

Two randomized trials demonstrated a benefit in quality of life for patients who received surgery plus wbrt as compared with patients who received wbrt alone^5^,^8^, and one randomized trial showed no significant difference between the groups^6^. In the trial by Patchell *et al.*^5^, the length of functional independence (defined as a kps ≥ 70), was significantly improved in the surgical group (8.8 months vs. 1.8 months, *p* < 0.005). Multivariate analysis showed that surgical treatment was the only factor associated with a better quality of life (*p* < 0.007). In the trial by Vecht *et al.*^8^, median functionally independent survival (defined in that trial as a World Health Organization performance status ≤ 1) was somewhat longer in patients who received surgery than in patients who received wbrt alone (7.5 months vs. 3.5 months, *p* = 0.06).

The analysis of patients with progressive extracranial disease demonstrated no difference in functionally independent survival between treatment groups (*p* = 0.88), but the analysis of patients with stable extracranial disease demonstrated a significant benefit for patients who received surgery as compared with patients who received wbrt alone (*p* = 0.01). In the trial by Mintz *et al.*^6^, no statistically significant differences were observed in the mean Spitzer quality-of-life score or the kps between treatment groups.

##### Adverse Effects

In the trials by Patchell *et al.*^5^ and Mintz *et al.*^6^, surgical mortality (defined as death within 30 days following surgery) did not differ significantly from 30-day mortality in the wbrt-only groups. In the trial by Vecht *et al.*^8^, 30-day mortality was 9% in the combined treatment group and 0% in the wbrt-only group; however, death within 2 months did not differ between the groups. In one trial^6^, 30-day morbidity was 8% in the surgery plus wbrt group and 17% in the wbrt-only group, and in another trial^6^, it did not differ between the groups.

In the trial by Vecht *et al.*^8^, postoperative complications included respiratory problems in 4 patients, intracerebral hemorrhage in 1 patient, infectious disease in 3 patients, and other complications in 9 patients. Postoperative morbidity affected 13 patients, and those complications were serious in 4 patients. Complications of radiotherapy, including nausea, vomiting, and headache, did not differ between the treatment groups (10 patients in the surgery plus wbrt group vs. 9 patients in the wbrt-only group). In the Cochrane meta-analysis, no significant difference in adverse effects was detected between the groups (or: 1.25; 95% ci: 0.68 to 2.66; *p* = 0.39)^9^.

#### 4.2.2 Should Patients with Single Brain Metastasis Undergoing Surgical Resection Receive Adjuvant WBRT?

Although all three rcts examining the efficacy of surgery for single brain metastasis also administered wbrt to the surgical treatment arm^5^,^6^,^8^, the need for postoperative wbrt had not been established through randomized trials. Patchell *et al.*^10^ conducted a follow-up rct comparing surgery plus wbrt with surgery alone to determine whether postoperative wbrt increases survival or the neurologic control of disease. The researchers randomly assigned 49 patients to postoperative wbrt and 46 patients to observation after complete resection of a single brain metastasis. Contrast-enhanced mri was performed after resection to confirm complete resection and to rule out additional lesions, and resected tissue was examined to confirm that all patients had metastatic tumours. Patients were required to have a kps of ≥70. Patients with small-cell lung cancer, germ-cell tumours, lymphoma, leukemia, or multiple myeloma were excluded, and the included patients were stratified by type and extent of extracranial disease.

Recurrence of a tumour at the site of the original metastasis (10% vs. 46%, *p* < 0.001) or anywhere else in the brain (18% vs. 70%, *p* < 0.001) was less frequent in the wbrt group than in the observation group^10^. Patients in the radiation group were less likely to die of neurologic causes than were patients in the observation group (14% vs. 44%, *p* = 0.003); however, no significant difference was observed in overall length of survival or length of time that patients remained functionally independent.

#### 4.2.3 What Is the Role of SRS in the Management of Patients with Single Brain Metastasis?

##### WBRT With or Without SRS

One rct compared the use of wbrt plus srs boost with wbrt alone in patients with brain metastases^11^. The Radiation Therapy Oncology Group 9508 rct by Andrews *et al.* randomized patients with 1–3 brain metastases, including 186 patients with a single metastasis, to receive either wbrt plus srs or wbrt alone. The target sample size was calculated to provide sufficient statistical power to detect a survival difference between treatment arms in patients with single brain metastasis.

Patients with a kps of <70, with lesions greater than 4 cm in diameter, or with known active extracranial disease were excluded from the study. Patients randomized to srs boost received srs within 1 week after wbrt. Fourteen patients with a single metastasis (15%) randomized to srs boost did not receive radiosurgery, but were included in the analysis in an intent-to-treat approach. As compared with patients receiving wbrt alone, patients receiving both wbrt and srs in cases of single brain metastasis showed a significant improvement in median survival (6.5 months vs. 4.9 months, *p* = 0.039). The causes of death and the rates of adverse effects did not differ between treatment groups. Local control and quality-of-life results were not reported separately for patients with a single brain metastasis.

##### SRS Versus Surgical Resection

No randomized trials compared srs with traditional surgical resection; however, three retrospective reviews compared those treatment modalities^18^,^19^,^21^.

The study by Muacevic *et al.*^18^ reviewed 108 patients with a single metastasis no larger than 3.5 cm in diameter and stable systemic disease who received srs alone or surgery plus wbrt. Patients in the srs group had significantly smaller tumours than did the patients in the surgery plus wbrt group (mean size: 2.07 cm vs. 2.7 cm; *p* < 0.001). The srs group also contained a higher proportion of patients with melanoma. Although median survival was 15.7 months in the surgery plus wbrt group and 8.1 months in the srs group, that survival difference was not statistically significant. No significant differences in local control or complications were observed between the groups, but a higher incidence of distant recurrences was reported in the srs group.

The review by Schöggl *et al.*^19^ retrospectively matched 133 patients who received wbrt and either GammaKnife (Elekta, Stockholm, Sweden) srs or surgery for the treatment of a single brain metastasis less than 3 cm in diameter. Median survival and 1-year overall survival did not differ significantly between the groups; however, the authors reported that srs was superior for local control and morbidity.

To be included in the review by O’Neill *et al.*^21^, patients had to be candidates for both srs and surgical resection. Tumour size had to be no larger than 3.5 cm in diameter, and patients with deep-seated tumours or ventricular obstruction were excluded. These inclusion criteria were met by 23 patients who had received srs and 74 patients who had received surgery, most of whom had also received wbrt. Significantly fewer patients in the srs group had a good performance score (*p* = 0.0016). No significant differences in survival or cause of death were detected between the groups, and the authors concluded that neither srs nor surgical resection was superior in that study.

No conclusions can be drawn from the results of the foregoing studies because of the inherent limitations associated with comparisons that use retrospective data. Those reviews were subject to selection bias, and the patients in the two groups differed in important prognostic factors such as performance status and tumour size. In addition, small sample sizes limited the ability of the studies to detect significant differences between treatment groups for key outcomes.

##### SRS With or Without WBRT

No randomized trials compared srs plus wbrt to srs alone; however, several retrospective reviews addressed the efficacy of srs with or without wbrt. A subgroup analysis of the largest review by Sneed *et al.*^20^ compared 168 patients with single brain metastasis who received srs alone with 175 patients who received srs with wbrt as initial treatment. To be included in the srs plus wbrt arm of the study, patients had to have received both radiosurgery and wbrt within a period of 1 month, although the order of treatment was not specified. Overall, patients who received srs alone included a higher percentage of patients more than 65 years old and with a kps of <70, but whether that imbalance was also present in patients with single metastasis is unclear. A number of patients, particularly those who initially received srs alone, underwent one or more salvage therapies for recurrence or new metastases. No significant survival difference was detected between the groups (Table III). Tumour control results were not reported for patients with single brain metastasis.

Flickinger *et al.*^15^ reviewed 116 patients with single metastasis treated with linear accelerator srs. Of those patients, 56% also received fractionated radiation therapy. In that study population, 45 patients (39%) had tumours that recurred after previous wbrt, and 71 (61%) were treated with srs as initial management for their metastasis. The median survival was 11 months, with local tumour control in 85% of patients. Recurrence was documented in 15%. In a multivariate analysis, local tumour control was significantly better in patients receiving both fractionated radiation therapy and srs as compared with srs alone (*p* = 0.011), but no effect on survival was observed.

Two non-comparative retrospective reviews^16^,^17^ and one single-arm prospective case series^12^ investigated the efficacy of srs plus wbrt. The study by Auchter *et al.*^17^ retrospectively reviewed 122 patients who matched the eligibility criteria for entry into the randomized trial by Patchell *et al.*^6^ and who had been treated with srs followed by wbrt. None of those patients had received prior surgery or radiation therapy. Median survival was 12.9 months, and the 1- and 2-year survival rates were 53% and 30% respectively. Complete response was observed in 25% of patients, and partial response in 34%. Local control rates at 1 and 2 years were 85% and 77% respectively. Intracranial recurrence outside the srs volume was experienced by 22% of patients. Median duration of functionally independent survival, defined as a kps > 70, was 10.2 months.

A second retrospective review by Alexander *et al.*^16^ included 171 patients with single brain metastasis. Most of the patients in that review received srs to treat recurrent lesions. All patients received wbrt, either as part of their initial therapy or in combination with srs. Median survival for patients with single brain metastasis was 10.3 months. A small prospective case series of 24 patients who received srs plus wbrt^12^ reported a median survival of 10 months and tumour shrinkage in 58% of patients for whom data were available.

Two single-arm prospective studies^13^,^14^ investigated the efficacy of srs alone. The case series by Sturm *et al.*^13^ of 30 patients with inoperable single brain metastasis reported mean survival of 6.5 months, improvement of clinical symptoms in 18 of 27 patients, and tumour regression in 13 of 22 patients. A subgroup analysis of the study by Lutterbach *et al.*^14^ reported median survival of 7.7 months for patients with single brain metastasis.

## 5. DISCUSSION

### 5.1 Should Patients with Confirmed Single Brain Metastasis Undergo Surgical Resection Before Radiation Therapy?

Definitive conclusions about using resection before radiation therapy are difficult to reach. The three rcts that compared surgery plus wbrt with wbrt alone were relatively small, and they varied with respect to important baseline patient characteristics. The largest trial by Mintz *et al.*^6^ was calculated to have only 50% statistical power to detect a 50% difference in median survival between treatment arms^22^. The two major differences between the results of the three rcts are the reduced survival time for the surgery plus wbrt group in the Mintz *et al.* rct^6^ and the diminished survival time reported by Patchell *et al.*^5^ for the wbrt-only group.

Several factors may have contributed to the reduced survival time for the wbrt-only group in the Patchell *et al.*^5^ trial.

Macdonald and Cairncross^23^ suggest that that trial may have had a referral bias. Patients in the trial were recruited from a cohort of patients referred to the neurosurgery service; thus, they represented a selected group of patients who were thought to be likely to benefit from surgery or who required more urgent surgery. Referral bias of that kind was minimized in the trial by Mintz *et al.*^6^, in which eligible patients were identified by oncologists, neurologists, and surgeons rather than being identified from among patients referred to the neurosurgery service.

Differences in the proportions of primary tumour histologies are another explanation for the lower survival for the radiation-only group in the Patchell *et al.*^5^ trial. That trial had a large proportion of patients with non-small-cell lung cancer (77.0%) as compared with the trials by Vecht *et al.* (52.3%)^8^ and Mintz *et al.* (53.6%)^6^. Because non-small-cell lung cancer is a relatively radioresistant tumour, the higher proportion of that tumour type may have biased the results against wbrt alone. Patchell *et al.* reported that lung cancer was not found to be a significant variable in a multivariate analysis of survival, but their small sample size may have had low statistical power to detect a difference.

The benefit of surgery may be lost in patients with poor prognostic factors such as advanced extracranial disease or lower performance status. Decreased median survival was reported in two randomized trials^6^,^8^ in patients with greater systemic involvement for their primary malignancy. Of the patients in the study by Mintz *et al.*^6^, 45% had extracranial metastases; in the trial by Patchell *et al.*^5^, this number was only 37.5%, and in the trial by Vecht *et al.*^8^, it was 31.7%. In the report by Mintz *et al.,* the univariate Cox regression model identified extent of disease as the most significant variable, with a relative risk of 1.86 (*p* = 0.006). Vecht *et al.* reported no difference in median survival for patients with progressive extracranial disease in the two groups; however, a significant survival advantage was reported for patients with stable disease who received surgery plus wbrt as compared with patients who received wbrt alone.

In the trial by Mintz *et al.*^6^, 21% of patients had a kps of <70, but patients in the trials by Patchell *et al.*^5^ and Vecht *et al.*^8^ had performance scores equivalent to a kps of ≥ 70. In addition, patients in the trials by Patchell *et al.* and Vecht *et al.* were required to have a minimum life expectancy of 6 months, but that expectation was not required by Mintz *et al.*^6^ in their trial. The increased proportion of patients with poor prognoses in the Mintz *et al.* trial, and the fact that 10 patients in the wbrt-only arm underwent a surgical procedure may have made it more difficult to detect a survival advantage for surgery.

A pooled analysis of the three trials showed no significant overall survival advantage for the surgical group as compared with the wbrt-only group^9^. However, the key differences in patient baseline characteristics between the studies and the wide confidence limits around the pooled estimate of effect allow for the possibility that surgery may have a beneficial effect on survival in selected groups of patients and may provide no survival benefit for others. The pooled results suggest that surgery may reduce mortality from neurologic causes, but in those studies, the difference with surgery was not statistically significant.

The evidence to determine whether surgical resection, as compared with treatment with wbrt alone, has a benefit on quality of life is limited. However, two rcts^5^,^8^ reported that surgery plus wbrt significantly prolonged functionally independent survival as compared with wbrt alone. The published meta-analysis reported no significant increase in adverse effects for patients who underwent surgical resection as compared with those who received wbrt alone.

Surgical excision should be considered for patients with prognostic factors that would increase the potential benefit of such aggressive treatment, because randomized trials have demonstrated a benefit in those patients. The applicable prognostic factors include good performance status, minimal or no evidence of extracranial disease, and a surgically accessible single brain metastasis amenable to complete excision. Because treatment in this disease is considered palliative, invasive local treatments must be individualized.

### 5.2 Should Patients with Single Brain Metastasis Undergoing Surgical Resection Receive Adjuvant WBRT?

The one randomized trial examining surgery plus wbrt versus surgery alone^10^ supports the use of post-operative wbrt. Tumour recurrence was significantly reduced at the original and distant sites alike, and patients were less likely to die of neurologic causes if radiation therapy was used postoperatively. However, no significant differences were observed in overall survival or in maintenance of functional independence between the two groups. The use of postoperative radiation is supported by that trial as a preventive for central nervous system relapse and neurologic death rather than as a contributor to survival time or maintenance of functional independence.

In its radiation component, the trial by Patchell *et al.*^10^ used 5040 cGy in 28 fractions, where the current standard management of patients with single brain metastasis in the United States is 3000 cGy in 10 fractions. The latter dosage is typically used in the standard arm of randomized studies of radiation in patients with brain metastases. Based solely on evidence, no reason exists to choose 3000 cGy in 10 fractions over 2000 cGy in 5 fractions, but fraction size is believed to be important and 300 cGy daily (3000/10) is believed to be associated with fewer long-term neurocognitive effects than is 400 cGy daily (2000/5) in the occasional long-term survivor, which is the reason that many radiation oncologists in Ontario prefer 3000 cGy in 10 fractions. Because no data exist to either support or refute that preference, there is no way to resolve this question of fractionation at present. More randomized trials examining various radiation therapy doses for patients with single brain metastasis are necessary to determine the optimal dose to maximize survival and minimize toxicity. The Neuro-oncology dsg will update the recommendations as new evidence becomes available.

### 5.3 What Is the Role of SRS in the Management of Patients with Single Brain Metastasis?

The randomized trial by Andrews *et al.*^11^ demonstrated a significant survival benefit for patients with single brain metastasis who received wbrt plus srs boost as compared with patients who received wbrt alone. With that evidence, it is reasonable to conclude that srs should be considered for patients with a small single brain metastasis, good performance status, and controlled extracranial disease who also meet additional eligibility criteria for srs.

The evidence comparing the efficacy of srs with that of surgery in the treatment of single brain metastasis is limited to retrospective reviews. Radiosurgery has been used increasingly in recent years because of minimal invasiveness, low risk, and ability to treat metastases considered surgically unresectable. No significant difference in survival was detected for patients receiving srs as compared with surgery in the three studies included in the present review^18^,^19^,^21^; however, one study suggested a benefit for srs in local control and morbidity^19^. Those studies were limited by small sample size and differences between treatment cohorts in key prognostic factors such as tumour size and performance status. Patients in those studies represent a highly selected study population, and the results therefore need to be interpreted cautiously. Preliminary evidence suggests a similar efficacy for srs and surgery, but direct comparisons using random patient allocation are needed to determine which treatment should be administered to patients who are candidates for both modalities.

The evidence comparing srs plus wbrt with srs alone is of poor quality and should be viewed only as hypothesis-generating. The addition of wbrt to srs has yet to be clarified through randomized trials. The rationale for using wbrt in addition to srs over srs alone is similar to the reasons presented for the use of radiation therapy following surgery. Use of wbrt allows for irradiation of any microscopic intracranial tumour deposits not revealed by neuro-imaging studies^24^ and metastases that have infiltrated into the brain beyond the srs margins. An additional theoretic consideration for using combined srs and wbrt relates to tumour shrinkage, which may occur after initial treatment with fractionated wbrt. The smaller radiosurgical target may provide better local control and reduced complication rates. Although the addition of wbrt to srs appears to increase local and distant intracranial control, wbrt may be associated with adverse effects such as radiation-induced dementia, particularly in long-term survivors. No quality data exist to help determine whether wbrt should be given before or after srs or whether selected patients should receive wbrt at recurrence or progression only.

A recent rct by Aoyama *et al.* 25 that compared srs plus wbrt with srs alone in patients with 1–4 brain metastases did not meet the inclusion criteria for the present systematic review because it did not separately report results for patients with single metastasis; however, 64 of 132 patients had single brain metastasis. The study did not detect a significant difference in overall survival between the treatment groups, but 1-year rates of brain tumour recurrence (46.8% vs. 76.4%, *p* < 0.001) and development of new brain metastases (41.5% vs. 63.7%, *p* = 0.003) were lower in patients who received srs plus wbrt than in those who received srs alone. Salvage treatment for brain tumour progression was required more frequently in patients who received srs alone than in those who received srs plus wbrt (*p* < 0.001).

The maximum size of lesions treatable with srs is not well established, although larger tumour volumes seem to be associated with poorer response and local control, and with higher complication rates. Radiosurgical treatment of larger metastases may increase the risk for development of necrotic lesions. Most studies included in the present review set limits for lesion diameter up to a maximum of 3 cm or 4 cm.

## 6. NEURO-ONCOLOGY DSG CONSENSUS PROCESS

The Neuro-oncology dsg decided to limit the target population for the present guideline by excluding patients with metastatic lymphoma, small-cell lung cancer, germ-cell tumour, leukemia, and sarcoma because these primary tumours are radiosensitive and respond differently to radiation therapy than do other tumours.

After reviewing the guideline report, the dsg members discussed the role of postoperative wbrt in terms of increased survival. Other issues addressed included srs versus surgical resection and the use of wbrt plus srs. The Neuro-oncology dsg drafted recommendations based on the evidence and attempted to draft recommendations based on perceived practice variations within Ontario.

## 7. EXTERNAL REVIEW OF THE PRACTICE GUIDELINE

### 7.1 Report Approval Panel Process

Before the present practice guideline was submitted for external review, the report was reviewed and approved by the pebc rap, which consists of two members, including an oncologist with expertise in clinical and methodology issues. Key issues raised by the rap included these points:

- For the first question on surgical resection, three small rcts were included. Analyses by both intent to treat and actual treatment received would be helpful. The dsg should indicate compliance with the assigned therapy and expand the interpretations in the Discussion section if the data demonstrate compliance problems.
- The subsection on quality of life cites only data regarding performance status. If these are the only data available, the dsg should consider renaming the section and address the topic as a performance-status outcome evaluation rather than as an assessment of quality of life.
- The dsg should consider a more definitive recommendation stating that the data are insufficient to recommend srs as single-modality therapy.

In response to this feedback, the Neuro-oncology dsg made the following modifications to the report and guideline:

- Information regarding treatment compliance was added to the Results section and to the Discussion of the systematic review. The three rcts did not perform analyses according to treatment received.
- The authors changed the title of the quality of life subsection to Quality of Life and Performance Status to reflect the performance status focus of most of the data.
- The dsg added a statement to the recommendations to emphasize that the evidence is insufficient to recommend srs as single-modality therapy.

### 7.2 Practitioner Feedback

#### 7.2.1 Methods

Practitioner feedback on the draft practice guideline report was obtained through an electronic survey of 98 practitioners in Ontario (medical oncologists, radiation oncologists, neurologists, and neurosurgeons). The survey consisted of items evaluating the methods, results, and discussion used to inform the draft recommendations and whether the draft recommendations should be approved as a practice guideline. Written comments were invited. Follow-up reminders were sent at 4 weeks and 6 weeks.

#### 7.2.2 Results

Of the 23 practitioners who responded, 16 indicated that the report was relevant to their clinical practice, and they completed the survey. The other 7 practitioners indicated that they were not able to complete the survey or that the report was not relevant to their clinical practice. These were the key results of the practitioner feedback survey:

Number surveyed: 98

Number of responses: 23 (23%)

Number who completed the survey: 16 (70%)

Written comments attached: 6 (38%)

Agreement with the summary of the evidence: 14 (88%)

Agreement with the recommendations: 12 (75%)

Approval of the recommendation as a practice guideline: 12 (75%)

#### 7.2.3 Summary of Main Findings

Of respondents who completed the survey, 6 (38%) provided written comments. The main points contained in the written comments are summarized below.

- One respondent commented that the draft was very good.
- One respondent stated that the recommendations were as clear as possible given the vagueness of the literature. This respondent felt that the data regarding surgical excision and adjuvant radiation were clear enough and agreed fully with this aspect of the report.
- One respondent commented that implementation of srs would require significant resource allocation.
- Two respondents stated that the rct by Aoyama *et al.*^25^ comparing srs plus wbrt with srs alone should be included in the systematic review. Although the full publication was outside the scope of the literature review, results were available in abstracts from the 2004 American Society of Clinical Oncology annual meeting.
- One respondent suggested that the study by Auchter *et al.*^17^ would be more appropriately included and discussed in the SRS Versus Surgical Resection subsection, because it describes a patient population that would have been eligible for surgery but that was treated with radiosurgery.
- One respondent commented that few high quality data support the statements made in the report and that the data comparing srs with surgery are as good as any of these. The limited evidence suggests that srs and surgery are roughly equivalent, and srs (with or without wbrt) should be strongly considered for single lesions that are not amenable to surgery. The report should mention this consideration.
- One respondent found it difficult to comment on the draft recommendations because they were not clear and because no definitive recommendation was made for each of the questions. The respondent suggested that a summary of the recommendations would be helpful.

#### 7.2.4 Modifications or Actions

Based on the practitioner feedback survey, the following actions were taken or modifications made:

- The text was amended to state that issues of cost of treatment and resource allocation are beyond the scope of this evidence-based guideline.
- The rct by Aoyama *et al.* 25 was still excluded from the systematic review because it did not report results for patients with single brain metastasis separately from those for patients with multiple metastases. A paragraph regarding this rct was added to the Discussion section of the systematic review.
- The study by Auchter *et al.*^17^ was a single-arm study of srs plus wbrt for patients whose tumours were considered resectable. This study was not included in the surgery versus srs subsection because it was not a comparative study and patients did not undergo surgical resection.
- The recommendations state that srs following wbrt should be considered for any patients with a tumour whose size and location are suitable for srs. The authors did not feel that a separate recommendation for srs specifically in patients with a single lesion not amenable to surgery was necessary.

## 8. PRACTICE GUIDELINE

The present practice guideline integrates the draft recommendations with the feedback obtained from the external review process. It has been approved by the Neuro-oncology dsg and the pebc rap.

### 8.1 Target Population

The recommendations that follow apply to adults with confirmed cancer and a single brain metastasis. This practice guideline does not apply to patients with metastatic lymphoma, small-cell lung cancer, germ-cell tumour, leukemia, or sarcoma.

### 8.2 Recommendations

Surgical excision should be considered for patients with good performance status, minimal or no evidence of extracranial disease, and a surgically accessible single brain metastasis amenable to complete excision. Because treatment in cases of single brain metastasis is considered palliative, invasive local treatments must be individualized. Patients with lesions requiring emergency decompression because of intracranial hypertension were excluded from the rcts, but should be considered candidates for surgery.

Postoperative wbrt should be considered to reduce the risk of tumour recurrence for patients who have undergone resection of a single brain metastasis. The optimal dose and fractionation schedule for wbrt is 3000 cGy in 10 fractions or 2000 cGy in 5 fractions.

As an alternative to surgical resection, wbrt followed by srs boost should be considered for patients with single brain metastasis. The evidence is insufficient to recommend srs alone as single-modality therapy.

### 8.3 Qualifying Statements

No high-quality data are available regarding the choice of surgery versus radiosurgery for single brain metastasis. In general, size and location of the metastasis determine the optimal approach.

The standard wbrt regimen for management of patients with single brain metastasis in the United States is 3000 cGy in 10 fractions, and this treatment is usually the standard arm in randomized studies of radiation in patients with brain metastases. Based solely on evidence, the understanding that no reason exists to choose 3000 cGy in 10 fractions over 2000 cGy in 5 fractions is correct; however, fraction size is believed to be important, and therefore 300 cGy daily (3000/10) is believed to be associated with fewer long-term neurocognitive effects than is 400 cGy daily (2000/5) in the occasional long-term survivor. For that reason, many radiation oncologists in Ontario prefer 3000 cGy in 10 fractions. No data exist to either support or refute that preference; therefore, finding a resolution to this issue is not currently possible. The Neuro-oncology dsg will update the recommendations as new evidence becomes available.

## 9. PRACTICE GUIDELINE DATE

Approved on August 16, 2006. Practice guidelines developed by Cancer Care Ontario’s pebc are regularly reviewed and updated. Please visit the Web site of Cancer Care Ontario’s pebc (www.cancercare.on.ca/index_AboutthePEBC.htm) for updates of this guideline.

## Figures and Tables

**TABLE I:** Studies included in the present systematic review

Question	Study type	Reference
1. Should patients with confirmed single brain metastases undergo surgical resection?	3 rcts 1 meta-analysis	5, 6, 8 9
2. Should patients with single brain metastases undergoing surgical resection receive adjuvant wbrt?	1 rct	10
3. What is the role of srs in the management of patients with single brain metastases?	1 rct 3 prospective case series 7 retrospective reviews	11 12–14 15–21

rct = randomized controlled trial; wbrt = whole-brain radiation therapy; srs = stereotactic radiosurgery.

**TABLE II:** Randomized trials of surgery plus radiation therapy as compared with radiation therapy alone

Reference	Treatment	Patients (n)	Eligibility criteria	Steroids	Median survival (months)	Local recurrence (%)	Median functionally independent survival (months)
Patchell *et al.* 1990^5^	wbrt wbrt + surgery	23 25	kps≥70, age≥18	All	3.5 9.2 *p<*0.01	52 20 *p<*0.02	1.8 8.8 *p<*0.005
Vecht *et al.* 1993^8^	wbrt wbrt + surgery	31 32	who ps≤2, age≥18	Most	6 10 *p=*0.04	nr	3.5 7.5 *p=*0.06
Mintz *et al.* 1996^6^	wbrt wbrt + surgery	43 41	kps≥50, age<80	All	6.3 5.6 *p=*0.24	nr	nr

wbrt = whole-brain radiation therapy; kps = Karnofsky performance status; who ps = World Health Organization performance status; nr = not reported.

**TABLE III:** Studies investigating stereotactic radiosurgery (srs)

Reference	Study type	Treatment	*Patients* (n)	Metastasis diameter (cm)	Median survival (months)	Comments
wbrt with or without srs						
Andrews *et al.* 2004^11^	rct	wbrt WBRT + SRS	94 92	≤4	4.9^a^ 6.5^a^ *p=*0.0393	Patients with prior surgery not excluded. Patients with active disease excluded. wbrt: 37.5 Gy in 15 fractions srs: 15–24 Gy, linac or GammaKnife^b^
srs versus surgical resection						
Muacevic *et al.* 1999^18^	rr	srs Surgery + wbrt	56 52	≤3.5	8.1 15.7	srs group: No surgery or wbrt. Surgical re-treatment not excluded from surgery group. wbrt: 40 Gy + 10-Gy boost. srs: 14–27 Gy, GammaKnife^b^.
Schöggl *et al.* 2000^19^	rr	srs + wbrt Surgery + wbrt	67 66	<3	12 9 *p=*0.55	Limited systemic disease. wbrt: 30 Gy in 10 fractions. srs: median 17 Gy, GammaKnife^b^
O’Neill *et al.* 2003^21^	rr	srs^c^ Surgery^c^	23 74	<3.5	13 16	No prior treatment. Patients are candidates for SRS and surgery. Patients with active systemic disease included.
srs with or without wbrt						
Sneed *et al.* 2002^20^	rr	srs srs + wbrt	168 175	nr	8.3 8.4 *p=*0.94	No prior surgery. srs + wbrt within 1 month. Some patients received salvage therapy >1 month after initial treatment. srs: linac or GammaKnife^b^
Coffey *et al.* 1991^12^	cs	srs + wbrt	24	≤3	10	3 patients received prior wbrt. srs: margin, 16–20 Gy; centre, 18–40 Gy; GammaKnife^b^
Auchter *et al.* 1996^17^	rr	srs + wbrt	122	nr	12.9	All metastases resectable. No prior treatment. wbrt: 25–40 Gy; fractions: 2–3 Gy. srs: 10–27 Gy (median: 17 Gy), linac.
Flickinger *et al.* 1994^15^	rr	srs +some wbrt	116	≤3.6	11	39% of patients treated for recurrent tumours following prior wbrt. 56% of patients received srs plus wbrt. srs: mean minimum dose, 17.9 Gy; mean maximum dose, 34.8 Gy; GammaKnife b.
Alexander *et al.* 1995^16^	rr	srs + wbrt	171	nr	10.3	srs: minimum dose, 9–25 Gy; maximum dose, 14–31.23 Gy.
Sturm *et al.* 1991^13^	cs	srs	30	nr	6.5^a^	All patients inoperable. srs: 20–30 Gy, linac.
Lutterbach *et al.* 2003^14^	cs	srs	55	≤3	7.7	No prior treatment. srs: 18 Gy, linac.

a Mean survival time.

b Elekta, Stockholm, Sweden.

c Most patients also received whole-brain radiation therapy (82% of surgery group and 96% of srs group).

rct = randomized controlled trial; wbrt = whole-brain radiation therapy; rr = retrospective review; nr = not reported; linac = linear accelerator; cs = case series.
